# The pathogenic S688Y mutation in the ligand-binding domain of the GluN1 subunit regulates the properties of NMDA receptors

**DOI:** 10.1038/s41598-020-75646-w

**Published:** 2020-10-29

**Authors:** Kristyna Skrenkova, Jae-man Song, Stepan Kortus, Marharyta Kolcheva, Jakub Netolicky, Katarina Hemelikova, Martina Kaniakova, Barbora Hrcka Krausova, Tomas Kucera, Jan Korabecny, Young Ho Suh, Martin Horak

**Affiliations:** 1 Department of Neurochemistry, Institute of Experimental Medicine of the Czech Academy of Sciences, Videnska 1083, 14220 Prague 4, Czech Republic; 2 Department of Biomedical Sciences, Neuroscience Research Institute, Seoul National University College of Medicine, 103 Daehak-ro, Jongno-gu, Seoul, 03080 South Korea; 3 Department of Physiology, Faculty of Science, Charles University in Prague, Albertov 6, 12843 Prague 2, Czech Republic; 4 Department of Military Medical Service Organisation and Management, and Department of Toxicology and Military Pharmacy; Faculty of Military Health Sciences, University of Defence, Trebesska 1575, 500 01 Hradec Kralove, Czech Republic; 5 Biomedical Research Centre, University Hospital Hradec Kralove, Sokolska 581, 500 05 Hradec Kralove, Czech Republic

**Keywords:** Molecular neuroscience, Ion channels in the nervous system

## Abstract

Although numerous pathogenic mutations have been identified in various subunits of *N*-methyl-D-aspartate receptors (NMDARs), ionotropic glutamate receptors that are central to glutamatergic neurotransmission, the functional effects of these mutations are often unknown. Here, we combined in silico modelling with microscopy, biochemistry, and electrophysiology in cultured HEK293 cells and hippocampal neurons to examine how the pathogenic missense mutation S688Y in the GluN1 NMDAR subunit affects receptor function and trafficking. We found that the S688Y mutation significantly increases the EC_50_ of both glycine and d-serine in GluN1/GluN2A and GluN1/GluN2B receptors, and significantly slows desensitisation of GluN1/GluN3A receptors. Moreover, the S688Y mutation reduces the surface expression of GluN3A-containing NMDARs in cultured hippocampal neurons, but does not affect the trafficking of GluN2-containing receptors. Finally, we found that the S688Y mutation reduces Ca^2+^ influx through NMDARs and reduces NMDA-induced excitotoxicity in cultured hippocampal neurons. These findings provide key insights into the molecular mechanisms that underlie the regulation of NMDAR subtypes containing pathogenic mutations.

## Introduction

*N*-methyl-D-aspartate receptors (NMDARs) are a subclass of ionotropic glutamate receptors that play an essential role in mediating excitatory neurotransmission and synaptic plasticity in the mammalian central nervous system (CNS)^1–3^. NMDARs are tetramers comprised of two GluN1 subunits (with eight splice variants) together with two GluN2 (GluN2A through GluN2D) and/or two GluN3 (GluN3A and GluN3B) subunits^3,4^. GluN2A and GluN2B are the principal GluN2 subunits expressed in the forebrain of mature animals^3–5^. NMDARs can also occur as triheteromeric GluN1/GluN2A/GluN2B^6–9^ or GluN1/GluN2/GluN3A^10–12^ receptors as well as diheteromeric GluN1/GluN3A receptors^13–16^. All GluN subunits share a basic membrane topological structure consisting of four membrane domains (M1 through M4), an intracellular C-terminal domain (CTD), an extracellular N-terminal domain composed of the amino-terminal domain and the S1 segment of the ligand-binding domain (LBD), and an extracellular loop between M3 and M4 containing the S2 segment of the LBD. GluN1/GluN2 receptors are gated by the simultaneous binding of an agonist (glutamate or NMDA) to the LBD in the GluN2 subunit and a co-agonist (glycine or  d-serine) to the LBD in the GluN1 subunit^3,17–19^. In contrast, GluN1/GluN3 receptors are gated by glycine binding to the LBD in the GluN3 subunit, with receptor desensitisation mediated by glycine binding to the LBD in the GluN1 subunit^20–23^. Thus, the LBD in the GluN1 subunit plays distinct functional roles in GluN1/GluN2 and GluN1/GluN3 receptors.

The number of NMDARs at the cell surface is regulated at multiple levels, including transcription/translation, processing in the endoplasmic reticulum (ER), trafficking to the cell surface, lateral diffusion through the membrane, and receptor internalisation, recycling, and degradation^3,4,24,25^. Most studies suggest that the intracellular CTD of GluN subunits plays a critical role in regulating the surface expression of NMDARs, including receptors that contain GluN2A and/or GluN2B subunits^5^. Interestingly, the extracellular part of mammalian GluN subunits is extremely large and contains the LBDs, which also play a critical role in regulating receptor trafficking^26^. For example, studies have shown that the structural integrity of the LBD in the GluN1 subunit of both GluN1/GluN2 and GluN1/GluN3 receptors^27,28^, as well as of the LBD in the GluN2B subunit of GluN1/GluN2B receptors^29^, are likely involved in the ER quality control of NMDARs. Furthermore, several studies found that specific agonists and co-agonists differentially regulate surface NMDARs; for example, d-serine regulates the mobility of GluN2A-containing NMDARs^30^, whereas glycine drives receptor internalisation^31^. However, precisely how structural changes in the LBD of GluN subunits regulate the surface delivery and function of specific NMDAR subtypes remains poorly understood.

Numerous pathogenic mutations have been identified in all GluN subunits‒encoding genes in patients with a variety of neuropsychiatric disorders and conditions^32–42^. The pathogenic mutations in GluN1 subunit have phenotypic similar with pathogenic mutations found in other GluN subunits, although they are usually associated with more sever course^43^. For example, the pathogenic mutations in GluN1 subunit were reported in patients with intellectual disability, cognitive dysfunction and development delay^43–48^, polymicrogyria^47^, oculomotor and movement disorders^43,45,46^, speech difficulties^43,45^, epilepsy^46–48^, postnatal microcephaly^47^, generalized cerebral atrophy^44^ or cortical blindness^47^. In addition, the pathogenic S688Y mutation in GluN1 subunit was associated with severe early infantile encephalopathy and some of the above symptoms^43^. Here, we used in silico modelling as well as electrophysiology, microscopy, and biochemistry in HEK293 cells and primary rat hippocampal neurons to examine the effect of the pathogenic S688Y mutation in the LBD of the GluN1 subunit on the trafficking and function of NMDARs. We found that the GluN1-S688Y subunit alters the functional properties of GluN1/GluN2A, GluN1/GluN2B, and GluN1/GluN3A receptors when expressed in both HEK293 cells and hippocampal neurons. Importantly, we also found that the S688Y mutation reduces surface delivery of GluN3A-containing NMDARs in both HEK293 cells and hippocampal neurons. Finally, we found that the S688Y mutation reduces both NMDA-induced Ca^2+^ influx and excitotoxicity in hippocampal neurons. Taken together, these findings reveal new insights in the role that this pathogenic mutation plays in regulating various NMDAR properties.

## Results

### The S688Y mutation in the GluN1 subunit causes a steric change in co-agonist binding

**Figure 1:**
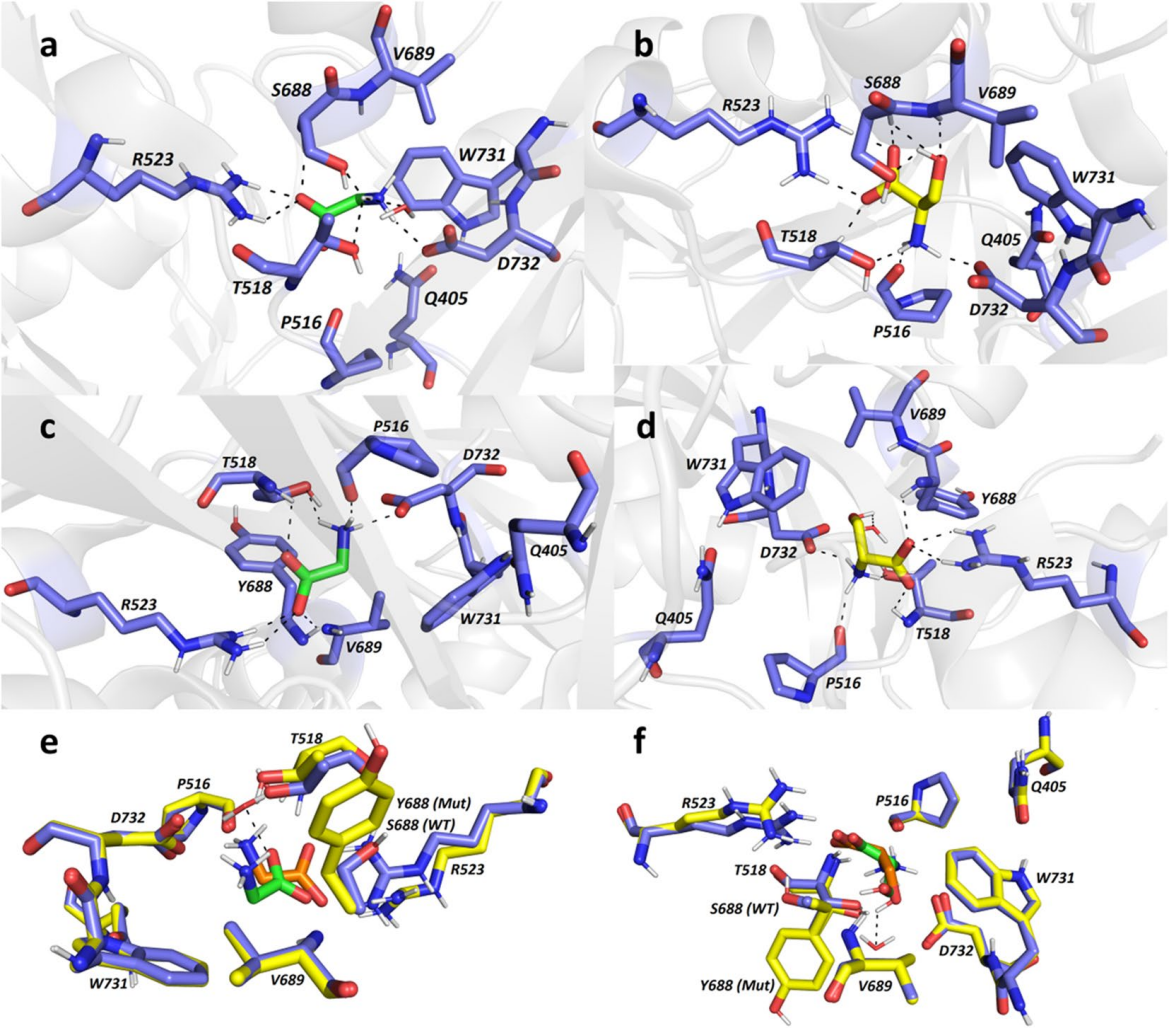
The S688Y mutation in the GluN1 subunit alters the binding of co-agonists in the receptor’s ligand-binding domain. (**a–d**) Glycine (in green; **a**,**c**) and  d-serine (in yellow; **b**,**d**) binding to the LBD of wild-type GluN1 (**a**,**b**) and GluN1-S688Y (**c**,**d**). The critical amino acid residues involved in co-agonist binding are shown as dark blue carbon atoms; hydrogen bonds are depicted as dashed lines, and the rest of the receptor is shown in light grey. (**e**,**f**) Superimposed structures of the LBD in wild-type (S688) GluN1 (shown as dark blue carbon atoms) and mutant (Y688) GluN1 (shown as yellow carbon atoms), with the glycine (**e**) and  d-serine (**f**) molecules shown in green (WT) and orange (Y688). The PyMOL Molecular Graphics System, Version 2.0.6, Schrödinger, LLC (https://pymol.org/2/) was used to make the figure.

Here, we examined the effects of the previously reported S688Y mutation in the LBD of the GluN1 subunit^43^, focusing our study on the function and trafficking of NMDARs in cultured HEK293 cells and primary hippocampal neurons. As a first step, we performed molecular modelling using an in silico model of the human GluN1/GluN2A receptor^49^ to compare the structural properties of glycine and  d-serine binding at the LBD between wild-type GluN1 and the GluN1-S688Y mutant subunit (Fig. 1). We first docked glycine at the LBD of wild-type GluN1, yielding an excellent root-mean-square deviation (RMSD) score of 0.332 Å, thus validating our approach. Consistently with crystallographic data^49^, glycine (shown in green in Fig. 1) binds via several hydrogen bonds and electrostatic interactions (Fig. 1a), including interactions between glycine’s carboxyl group and the guanidinium moiety in R523^50^, the backbone amide groups in T518 and S688, and the hydroxyl group in S688. In addition, the glycine molecule’s positively charged ammonium group forms bonds with the carboxylate in D732, the hydroxyl groups in T518 and S688, and one water molecule. In contrast with the reported crystal structure of glycine bound to the LBD in wild-type GluN1 subunit^49^, our model suggests that the carboxyl group in P516 is 4.7 Å from the glycine molecule; thus, a hydrogen bond between glycine and this residue is unlikely. Moreover, both W731 and Q405 are located relatively close to the glycine molecule and are presumably involved in other electrostatic interactions and/or water-mediated bridges, although this is difficult to estimate using docking studies. With respect to the interaction between  d-serine (shown in yellow in Fig. 1b,d) and the LBD in wild-type GluN1, our model suggests that the  d-serine molecule likely forms hydrogen bonds between its functional carboxyl group and the guanidinium moiety in the R523 residue and the backbone amides in S688 and T518 (Fig. 1b). In addition, the  d-serine molecule’s hydroxyl group is located near the backbone amides in V689 and S688 and the hydroxyl group in S688, and the  d-serine molecule’s positively charged ammonium moiety forms contacts with the carboxylate group in D732, the carbonyl oxygen in P516, and the hydroxyl group in T518. Finally, our docking model did not reveal any direct interaction between  d-serine and a water molecule.

With respect to the GluN1-S688Y subunit, we found that glycine binding is generally similar to the wild-type GluN1 subunit (Fig. 1c); the only apparent difference was within the vicinity of the Y688 residue, in which the S688Y mutation shifted the glycine molecule toward the vicinity of the carbonyl oxygen in P516 residue and away from the V689 residue. Interestingly, the hydrogen bond formed between the glycine molecule’s carboxyl group and the backbone amide in residue 688 residue was unaffected by the mutation. Importantly, hydroxyl group of Y688 is too far away from glycine to form the hydrogen bond; the interaction between positively charged ammonium moiety in glycine and the aromatic region of Y688 residue is the only that can be observed. In contrast with glycine, we found considerable differences in  d-serine binding between the GluN1-S688Y and wild-type GluN1 subunits (Fig. 1d). Specifically, we found that the S688Y mutation appears to position the  d-serine molecule far from the V689 residue; thus, no hydrogen bond is formed between the ligand’s hydroxyl group and the backbone amide in V689. Interestingly, we also found that a water molecule plays a role in anchoring the  d-serine molecule’s hydroxyl group to the GluN1-S688Y subunit. The most striking finding is that the interaction between the  d-serine molecule and the GluN1-S688Y subunit lacks the hydrogen bond between the backbone amide in V689 residue and the ligand’s hydroxyl moiety, as well as the hydroxyl-hydroxyl interaction between  d-serine and residue 688. To help visualise the structural changes induced by the S688Y mutation, we superimposed the glycine-bound (Fig. 1e) and  d-serine‒bound (Fig. 1f) structures of the wild-type and mutant GluN1 LBDs. Glycine-bound and  d-serine-bound complexes generated a high degree of similarity with RMSD value of 0.140 Å.

In summary, our in silico modelling reveals that the presence of the more sterically demanding tyrosine at position 688 in the mutant GluN1 subunit decreases the apparent affinity of both glycine and  d-serine for binding the LBD.

### The GluN1-S688Y mutation alters the functional properties of human GluN1/GluN2A, GluN1/GluN2B, and GluN1/GluN3A receptors

**Figure 2:**
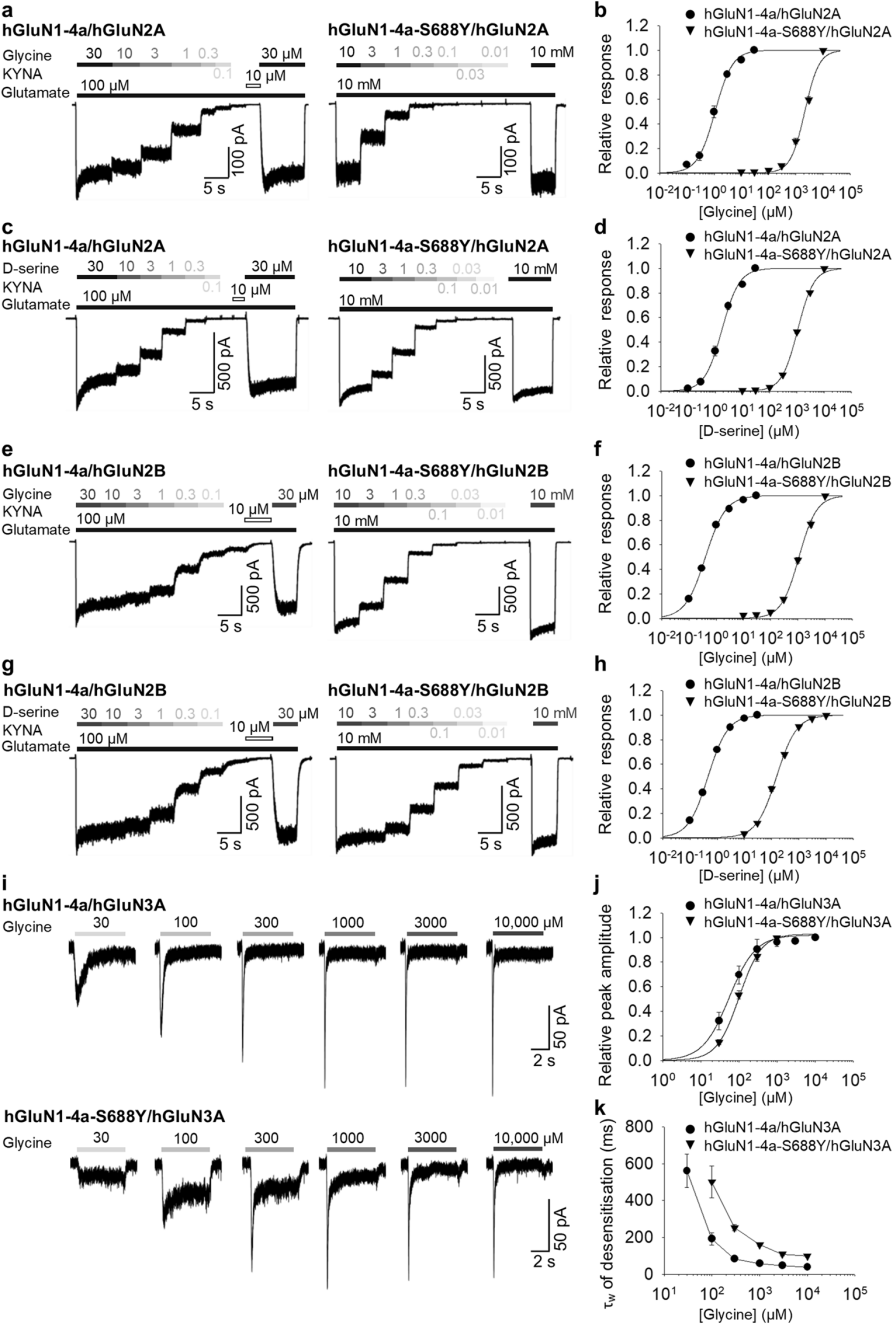
The S688Y mutation in GluN1 alters the receptor’s ligand affinity and desensitisation properties. (**a**,**c**,**e**,**g**) Representative whole-cell patch-clamp recordings of HEK293 cells co-transfected with hGluN1-4a and hGluN2 (left) or hGluN1-4a-S688Y and hGluN2 (right). Currents were elicited by applying glutamate (100 µM or 10 mM) and either glycine or  d-serine at the indicated concentrations; where indicated, the NMDAR antagonist 7-chlorokynurenic acid (KYNA, 10 µM) was applied. (**b**,**d**,**f**,**h**) Normalised steady-state concentration–response curves for cells expressing the indicated NMDAR subunits. Each data point represents the mean normalised steady-state current (± SEM). The EC_50_ value, Hill coefficient, and number of cells recorded for each group are listed in Table 1. (**i**) Representative whole-cell patch-clamp recordings of HEK293 cells co-expressing hGluN1-4a and hGluN3A (top) or hGluN1-4a-S688Y and hGluN3A (bottom). Currents were elicited by applying glycine at the indicated concentrations (µM). (**j**) Normalised peak concentration–response curves for cells expressing the indicated NMDAR subunits. Each data point represents the mean normalised peak current (± SEM). The EC_50_ value, Hill coefficient, and number of cells recorded for each group are listed in Table 2. (**k**) Summary of the τ_w_ of desensitisation measured in response to glycine in cells expressing the indicated NMDAR subunits.

To test our hypothesis that the S688Y mutation in GluN1 decreases the receptor’s affinity for glycine and  d-serine, we performed whole-cell patch-clamp recordings in HEK293 cells expressing human (h) GluN1/GluN2A, GluN1/GluN2B, and GluN1/GluN3A receptors. Specifically, we co-expressed either wild-type or hGluN1-4a-S688Y together with hGluN2A (Fig. 2a,c), hGluN2B (Fig. 2e,g), or hGluN3A subunits (Fig. 2i).

First, we generated concentration–response curves for glycine (Fig. 2b,f) and  d-serine (Fig. 2d,h) in the presence of 100 µM glutamate, as described previously^51^. The results are summarised in Table 1 and are consistent with previously reported EC_50_ values for glycine  d-serine^51^. To generate concentration–response curves for receptors containing the GluN1-S688Y subunit, we measured the responses in the presence of 10 mM glutamate, because the GluN1-S688Y mutation might affect the cooperativity among the LBDs within the functional NMDAR heterotetramer (see Supplementary Fig. S1). As shown in Fig. 2 and summarised in Table 1, the S688Y mutation caused a significantly reduced potency for both glycine and  d-serine, reflected by significantly increased EC_50_ values. Taken together, these findings support our modelling data, confirming that the S688Y mutation reduces the receptor’s affinity for both glycine and  d-serine.

**Table 1:** Summary of the fitting parameters for the steady-state concentration–response curves measured in HEK293 cells expressing the indicated NMDAR subunits (see Fig. 2b,d,f,h).

	hGluN1-4a/hGluN2A	hGluN1-4a-S688Y/hGluN2A	hGluN1-4a/hGluN2B	hGluN1-4a-S688Y/hGluN2B
**Glycine**				
EC_50_ (μM)^a^	1.09 ± 0.14	2122.78 ± 157.36*	0.41 ± 0.02	1100.09 ± 69.25*
*h* ^a^	1.37 ± 0.05	1.67 ± 0.07	1.18 ± 0.05	1.34 ± 0.07
*n*	7	9	8	6
**D-Serine**				
EC_50_ (μM)^a^	1.77 ± 0.19	1066.55 ± 29.65*	0.47 ± 0.02	152.09 ± 9.67*
*h*^a^	1.37 ± 0.03	1.48 ± 0.02	1.20 ± 0.03	1.19 ± 0.03
*n*	6	6	5	8

^a^The EC_50_ (in μM) and Hill coefficient (h) were obtained as described in the "Methods".

*p < 0.05 vs. the corresponding hGluN1-4a group (Student’s t-test).

Unlike GluN2-containing receptors, GluN3-containing receptors are activated by glycine binding to the LBD in the GluN3A subunit and desensitised by glycine binding to the LBD in GluN1^20–23^. We therefore measured currents induced by glycine at concentrations ranging from 30 µM to 10 mM in HEK293 cells expressing either hGluN1-4a/hGluN3A or hGluN1-4a-S688Y/hGluN3A receptors (Fig. 2i) and then analysed the peak concentration–response curve and the time constant for desensitisation, as described previously^27^. We found that cells expressing hGluN1-4a-S688Y/hGluN3A receptors were significantly less responsive to glycine compared to cells expressing wild-type receptors (Fig. 2j), with an ~ twofold decrease in glycine potency (Table 2). In addition, we found that hGluN1-4a-S688Y/hGluN3A receptors had an increased time constant (τ_w_) of desensitisation compared to wild-type receptors (Fig. 2k). Thus, although GluN1-S688Y subunits are capable of forming functional GluN1/GluN2 and GluN1/GluN3A receptors, its presence significantly alters the receptor’s functional properties.

**Table 2:** Summary of the fitting parameters for the peak concentration–response curves measured in HEK293 cells expressing the indicated NMDAR subunits (see Fig. 2j).

	hGluN1-4a/ hGluN3A	hGluN1-4a-S688Y/hGluN3A
**Glycine**		
EC_50_ (μM)^a^	55.49 ± 13.35	101.39 ± 9.98*
*h*^a^	1.26 ± 0.24	1.49 ± 0.10
*n*	5	5

^a^The EC_50_ (in μM) and Hill coefficient (h) were obtained as described in the "Methods".

*p < 0.05 vs. the corresponding hGluN1-4a group (Student’s t-test).

### The S688Y mutation in GluN1 differentially regulates the surface delivery of GluN1/GluN2 and GluN1/GluN3 NMDARs in HEK293 cells

**Figure 3:**
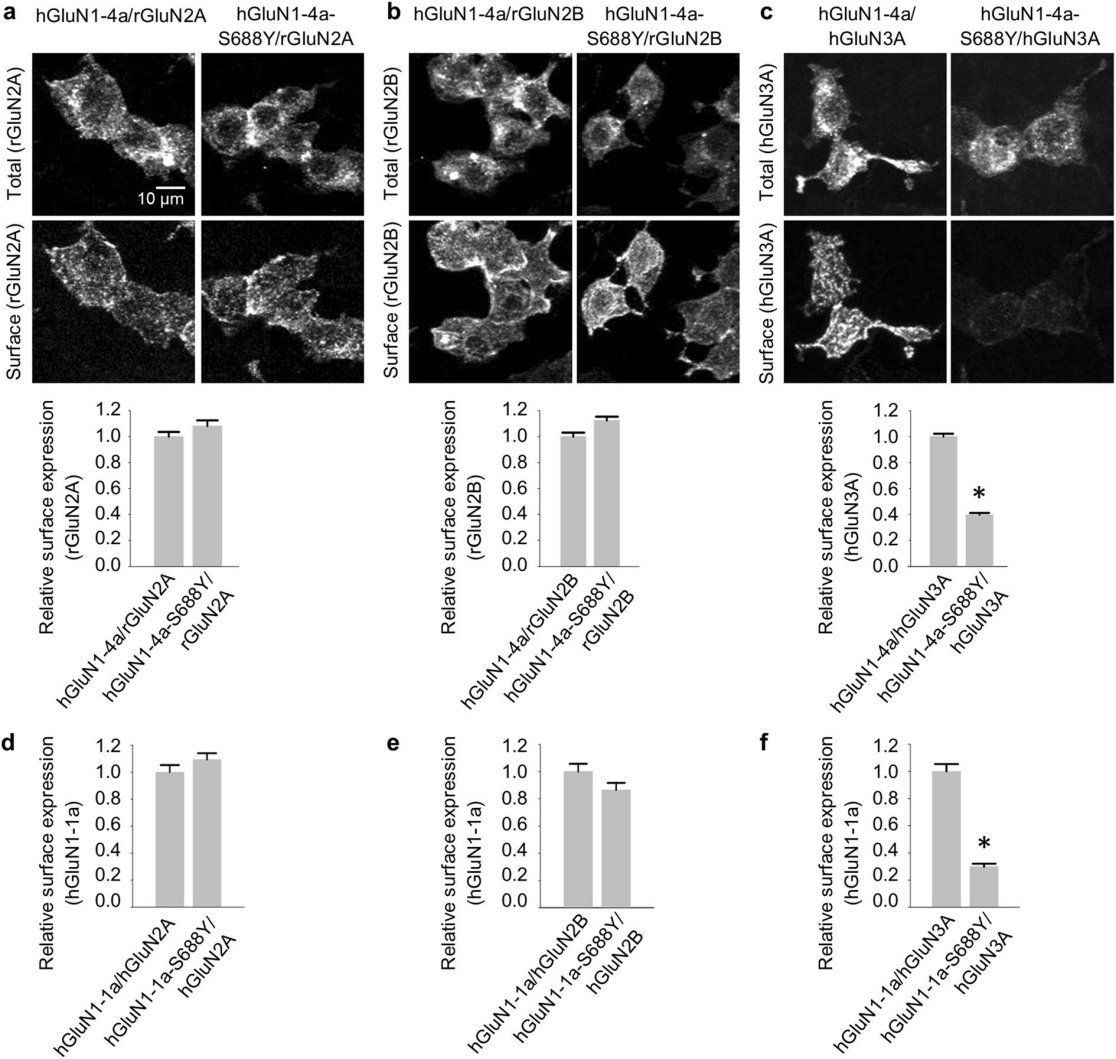
The S688Y mutation in GluN1 differentially regulates the surface expression of NMDAR subtypes in HEK293 cells. (**a–c**) Representative images of total and surface GFP-rGluN2A (**a**), GFP-rGluN2B (**b**), and GFP-hGluN3A (**c**) subunits measured in HEK293 cells lacking or expressing the indicated hGluN1-4a subunits. Shown below is the relative surface expression of GFP-rGluN2A, GFP-rGluN2B, and GFP-hGluN3A measured using fluorescence microscopy (*n* ≥ 192 cells per group); *p < 0.05 (one-way ANOVA followed by Dunnett’s post hoc test). (**d–f**) Summary of the relative surface expression of YFP-hGluN1-1a or YFP-hGluN1-1a-S688Y subunits expressed alone or together with hGluN2A, hGluN2B, and hGluN3A measured using fluorescence microscopy (*n* ≥ 125 cells per group); *p < 0.05 (one-way ANOVA followed by Dunnett’s post hoc test).

The D732A mutation in the glycine-binding site of GluN1 has been reported to reduce trafficking of GluN1/GluN2A receptors to the cell surface^28^; similarly, the integrity of the glutamate-binding site in GluN2B has been shown to regulate the trafficking of GluN1/GluN2B receptors^29^. We recently reported that the surface delivery of GluN1/GluN3A receptors is regulated by structural features in the glycine-binding sites of both GluN1 and GluN3A^27^. Here, we examined the effect of the S688Y mutation on the surface delivery of NMDARs expressed in HEK293 cells. To monitor expression, we co-transfected cells with either wild-type or hGluN1-4a-S688Y together with GFP-tagged rat GluN2A (GFP-rGluN2A), GFP-tagged rat GluN2B (GFP-rGluN2B), or GFP-tagged human GluN3A (GFP-hGluN3A; Fig. 3a–c). We then measured relative surface expression of the various NMDARs using fluorescence confocal microscopy. We found that the S688Y mutation did not affect the surface delivery of receptors containing either GFP-rGluN2A or GFP-rGluN2B subunits compared to the corresponding wild-type GluN1 subunits; in contrast, the mutation significantly reduced the surface delivery of receptors containing GFP-hGluN3A subunit (Fig. 3a–c). Similar results were obtained when we expressed the YFP-tagged hGluN1-1a-S688Y subunits together with hGluN2A, hGluN2B, or hGluN3A subunits compared to wild-type YFP-hGluN1-1a (Fig. 3d–f). Together, these data indicate that the S688Y mutation in GluN1 differentially regulates the surface expression of NMDARs in a subunit-dependent manner.

### The S688Y mutation in GluN1 reduces the surface delivery of GluN3A-containing subunits in hippocampal neurons

**Figure 4:**
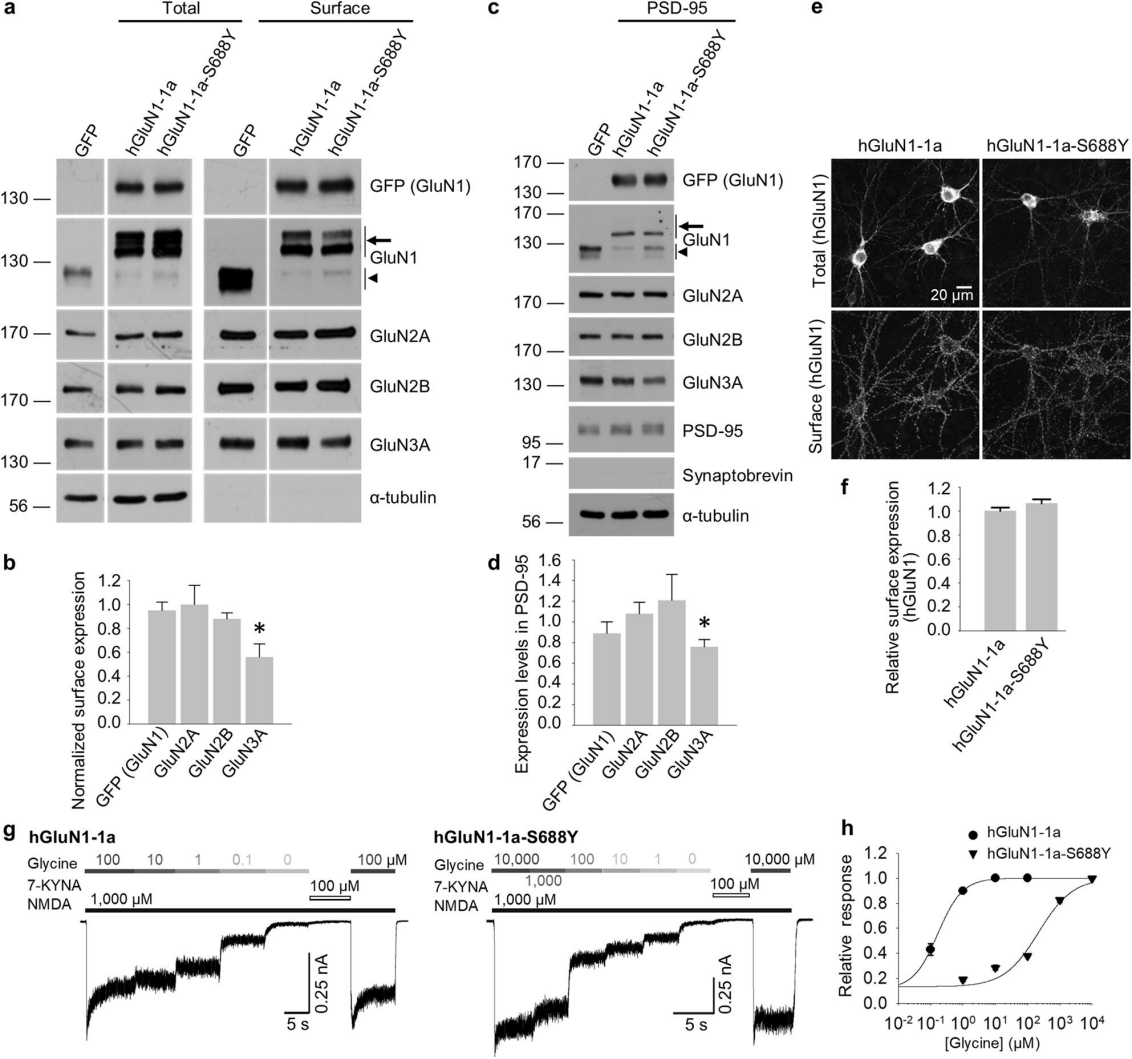
The S688Y mutation in GluN1 alters the surface expression of GluN3A-containing NMDARs and alters the ligand affinity of NMDARs in hippocampal neurons. (**a**) Cell-surface biotinylation assay of dense cultures of hippocampal neurons co-expressing the GluN1 shRNA together with YFP-hGluN1-1a or YFP-hGluN1-1a-S688Y. Surface NMDARs were biotinylated and then pulled down using streptavidin-agarose beads. Total input (2.5% of the lysate) and surface NMDAR subunits were then detected using the indicated antibodies by western blot analysis; the GFP antibody was used to detect the YFP-labelled GluN1 subunit. In the GluN1 blot, the arrow indicates the recombinant hGluN1-1a and hGluN1-1a-S688Y subunits, and the arrowhead indicates the endogenous GluN1 subunit. All biochemical results shown in (**a**) were cropped from the same blots and exposures for each antibody labelling (GFP, GluN1, GluN2A, GluN2B, GluN3A and α-tubulin; see Supplementary Fig. S2). (**b**) Summary of the relative surface expression of the indicated NMDAR subunits in neurons expressing YFP-hGluN1-1a or YFP-hGluN1-1a-S688Y. For each subunit, the ratio of surface to total band intensity was measured and normalised to the corresponding hGluN1-1a‒expressing group (*n* = 6 each); *p < 0.05 (Student’s *t*-test). (**c**) Western blot analysis of NMDAR subunits in the postsynaptic density (PSD) isolated from cortical neurons expressing the GluN1 shRNA together with YFP-hGluN1-1a or YFP-hGluN1-1a-S688Y. The positive and negative signals for PSD-95 and synaptobrevin, respectively, confirm that the samples contain the PSD fraction (see Supplementary Fig. S2). (**d**) Summary of the relative expression of the indicated NMDAR subunits in the PSD fraction, normalised to the corresponding hGluN1-1a‒expressing group (*n* = 5 each); *p < 0.05 (Student’s *t*-test). (**e**) Representative images of hippocampal neurons infected with YFP-hGluN1-1a or YFP-hGluN1-1a-S688Y, showing total and surface GFP immunostaining. (**f**) Summary of the relative surface expression of YFP-hGluN1-1a and YFP-hGluN1-1a-S688Y in hippocampal neurons measured from a 10-µm^2^ region of interest in secondary and tertiary dendrites (*n* ≥ 55 segments from ≥ 11 cells); *p < 0.05 (Student’s *t*-test). (**g**) Representative whole-cell patch-clamp recordings of primary hippocampal neurons expressing YFP-hGluN1-1a or YFP-hGluN1-1a-S688Y. Currents were elicited by applying glycine at the indicated concentrations in the presence of 1000 µM NMDA; where indicated, 10 µM KYNA was applied. (**h**) Steady-state concentration–response curves for hippocampal neurons expressing YFP-hGluN1-1a or YFP-hGluN1-1a-S688Y; each data point represents the mean normalised steady-state current (± SEM). The EC_50_ value, Hill coefficient, and number of cells recorded for each group are listed in Table 3.

Next, we examined whether the S688Y mutation in GluN1 also affects the surface delivery of GluN3-containing NMDARs in hippocampal neurons. Because the endogenous GluN1 subunit is robustly expressed in neurons^52^, we used shRNA to knock down endogenous GluN1 while expressing shRNA-resistant YFP-hGluN1-1a or YFP-hGluN1-1a-S688Y subunits, similarly as we employed previously^53^. We found that neurons expressing either YFP-hGluN1-1a or YFP-hGluN1-1a-S688Y had similar levels of both total and surface hGluN1 subunits, as well as similar levels of endogenous GluN2A and GluN2B subunits (Fig. 4a,b). In contrast, and consistent with our findings with HEK393 cells, we found that neurons expressing the YFP-hGluN1-1a-S688Y subunit had reduced levels of surface GluN3A compared to neurons expressing YFP-hGluN1-1a (Fig. 4a,b). Similar results were obtained when we examined GluN subunits in the postsynaptic density (PSD) fraction isolated from cortical neurons expressing YFP-hGluN1-1a or YFP-hGluN1-1a-S688Y (Fig. 4c,d). As additional confirmation, we used confocal microscopy to measure YFP-hGluN1-1a and YFP-hGluN1-1a-S688Y subunits in hippocampal neurons, finding similar surface expression (Fig. 4e,f). We also used electrophysiology to measure glycine-induced currents (in the presence of 1 mM NMDA) in hippocampal neurons expressing either YFP-hGluN1-1a or YFP-hGluN1-1a-S688Y (Fig. 4g). Consistent with our previous findings, we found that neurons expressing the YFP-hGluN1-1a-S688Y subunit have a significantly shifted concentration–response curve for glycine, with an EC_50_ of 220 µM compared to 0.2 µM for neurons expressing the YFP-hGluN1-1a subunit (Fig. 4h). Taken together, these results indicate that in hippocampal neurons, the S688Y mutation in GluN1 reduces the surface delivery of GluN3A-containing NMDARs, but not GluN2A- or GluN2B-containing receptors, and alters the receptor’s glycine affinity.

**Table 3:** Summary of the fitting parameters for the steady-state concentration–response curves measured in hippocampal neurons expressing the indicated YFP-hGluN1 subunits (see Fig. 4).

	hGluN1-1a	hGluN1-1a-S688Y
**Glycine**		
EC_50_ (μM)^a^	0.17 ± 0.02	218.62 ± 6.11*
*h*^a^	1.15 ± 0.05	0.82 ± 0.06
*N*	4	5

^a^The EC_50_ (in μM) and Hill coefficient (h) were obtained as described in the "Methods".

*p < 0.05 vs. the corresponding YFP-hGluN1-1a group (Student’s t-test).

### The S688Y mutation reduces NMDA-induced Ca^2+^ influx and excitotoxicity in hippocampal neurons

**Figure 5:**
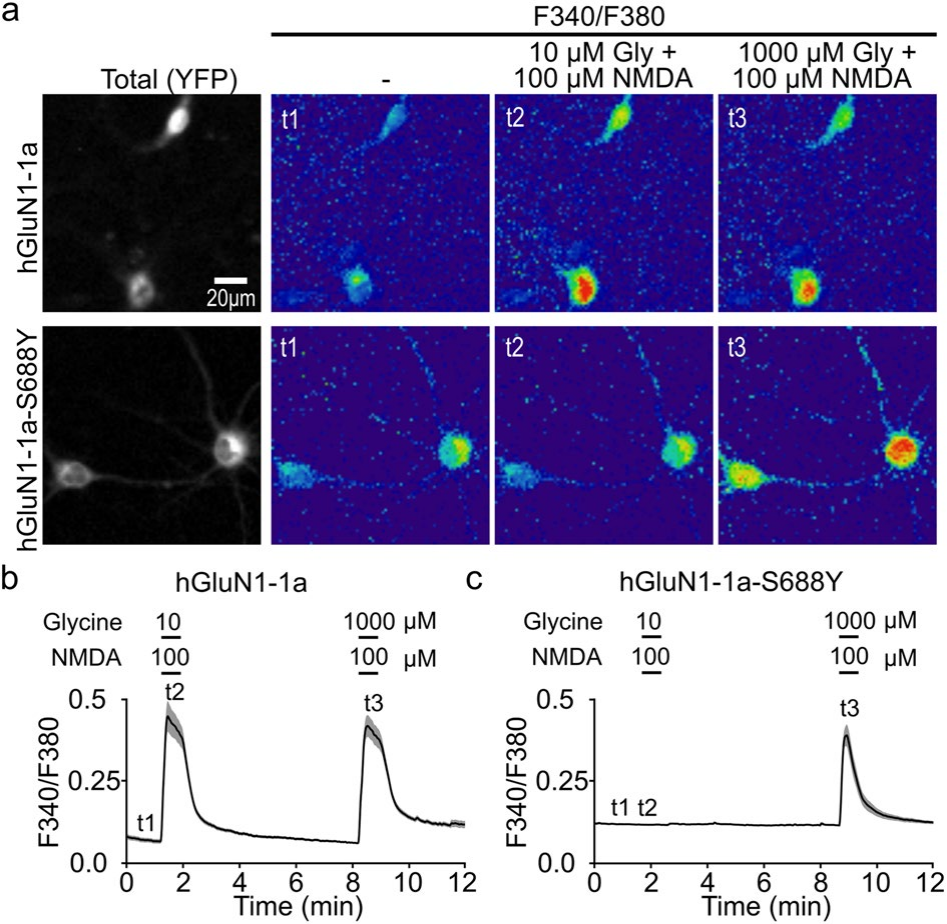
The S688Y mutation in GluN1 reduces NMDA-induced Ca^2+^transients in hippocampal neurons. (**a**) The intracellular Ca^2+^ was measured using the Ca^2+^ indicator Fura-2 in hippocampal neurons expressing YFP-hGluN1-1a (upper row) or YFP-GluN1-1a-S688Y (bottom row). The representative F340/380 ratiometric images show ratio at resting time (t1) and after application of 100 μM NMDA together with 10 µM (t2) or 1000 μM (t3) glycine as indicated. The cells appear red in accordance with raised Ca^2+^ levels. Below are shown averaged traces (± SEM) of the F340/F380 ratio measured from n ≥ 45 neurons per group for YFP-hGluN1-1a (**b**) and YFP-GluN1-1a-S688Y (**c**). Horizontal bars indicate applications (30 s duration) and the times t1-t3 correspond with the ratiometric images in (**a**).

Excessive influx of Ca^2+^ through NMDARs can induce neuronal cell death^54^. To examine whether the S688Y mutation in GluN1 alters the cell’s susceptibility to excitotoxicity, we measured intracellular Ca^2+^ in neurons expressing either YFP-hGluN1-1a or YFP-hGluN1-1a-S688Y subunits. Neurons were loaded with the Ca^2+^ indicator Fura-2 and then stimulated with either 10 or 1000 µM glycine together with 100 µM NMDA (Fig. 5a). We found that both 10 and 1000 µM glycine induced a robust increase in intracellular Ca^2+^ in cells expressing YFP-hGluN1-1a (Fig. 5a,b); in contrast, 1000 µM glycine induced a similar Ca^2+^ transient in YFP-hGluN1-1a-S688Y, whereas 10 µM glycine had no measurable effect on intracellular Ca^2+^ (Fig. 5a,c). These results support the notion that receptors containing GluN1-S688Y subunits have reduced NMDA-induced Ca^2+^ currents when expressed in hippocampal neurons.

**Figure 6:**
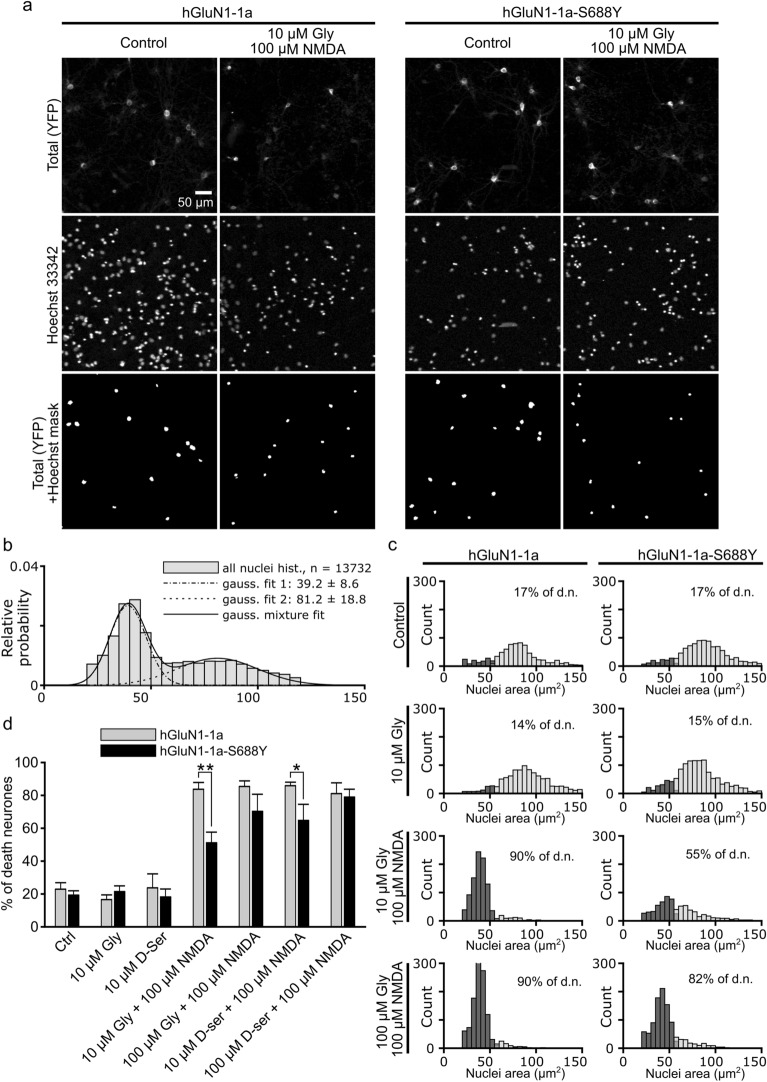
The S688Y GluN1 reduces NMDA-induced excitotoxicity in hippocampal neurons. (**a**) Hippocampal neurons expressing YFP-hGluN1-1a or YFP-hGluN1-1a-S688Y were treated with control solution or 100 µM NMDA together with 10 µM glycine 1 h; 23 h later, YFP-positive cells were analysed for excitotoxicity by staining with Hoechst 33,342. Shown are representative images. (**b**) The distribution of nuclear area reveals two distinct Gaussian peaks corresponding to pyknotic and non-pyknotic cells. The histogram was fitted with a 2-Gaussian distribution model (solid line), and the estimated individual Gaussian distributions are plotted as a dashed line (pyknotic cells) and a dotted line (non-pyknotic cells). (**c**) Distributions of nuclei areas measured for cells expressing YFP-hGluN1-1a or YFP-hGluN1-1a-S688Y and treated as indicated. All nuclei were classified as either pyknotic (dark grey bars) or non-pyknotic (light grey bars) group. (**d**) Summary of neuronal cell death (calculated as described in the "Methods") measured in cells expressing YFP-hGluN1-1a or YFP-hGluN1-1a-S688Y and treated as indicated (*n* ≥ 3269 cells per condition from 4 independent experiments); *p < 0.05 and **p < 0.01 (one-way ANOVA supported by the Shapiro–Wilk test of normality at *p* < 0.05)*.*

Finally, we examined whether the reduced Ca^2+^ influx in GluN1-S688Y‒containing receptors has physiologically relevant consequences by expressing either YFP-hGluN1-1a or YFP-hGluN1-1a-S688Y in hippocampal neurons and treating the cells for 1 h with 100 µM NMDA together with glycine (10 or 100 µM) or  d-serine (10 or 100 µM). Excitotoxicity was measured 23 h after treatment by staining cells with the nuclear marker Hoechst 33,342 and then performing complex image analysis (Fig. 6a,b; see "Methods"). We found that treating cells with control solution, 10 µM glycine alone, or 10 µM  d-serine alone induced only a small degree of cell death in neurons expressing either YFP-hGluN1-1a or YFP-hGluN1-1a-S688Y subunits (Fig. 6c,d). In contrast, treating YFP-hGluN1-1a‒expressing neurons with 10 µM glycine or 10 µM  d-serine in the presence of 100 µM NMDA caused nearly 100% excitotoxicity, consistent with previous reports^55^; similar results were obtained with YFP-hGluN1-1a‒expressing neurons treated with 100 µM glycine or 100 µM  d-serine in the presence of 100 µM NMDA (Fig. 6c,d). Importantly, however, YFP-hGluN1-1a-S688Y‒expressing neurons were less sensitive to NMDA-induced excitotoxicity, with significantly reduced cell death when treated with either 10 µM glycine or 10 µM  d-serine in the presence of 100 µM NMDA (Fig. 6c,d).

## Discussion

Here, we focused our study on NMDARs in the mammalian CNS, as these receptors play an important role in a wide range of physiological processes such as learning and memory^56,57^, as well as neuropathological processes such as neurodegeneration^54^. Specifically, we characterised the functional effects of the pathogenic S688Y mutation in the ligand-binding domain of the GluN1 NMDAR subunit^43^ using *in silic*o modelling and microscopic, biochemical, and electrophysiological analyses in HEK293 cells and rat hippocampal neurons.

Our experimentally measured EC_50_ values for both glycine and  d-serine with respect to wild-type GluN1/GluN2 receptors expressed in HEK293 cells are similar to previously published values for receptors expressed in *Xenopus* oocytes^3,58,59^. Moreover, we found that expressing the GluN1-S688Y subunit significantly decreased the receptor’s affinity for both glycine and  d-serine, with a more profound effect on glycine affinity. These findings are consistent with previous data showing that the S688A mutation produced a four-fold reduction in glycine potency^60^, the notion that the S688 residue in GluN1 plays a key role in ligand recognition^61^, as well as with our in silico modelling, which showed that the predicted interaction between the LBD in GluN1-S688Y differs slightly for glycine compared to  d-serine. In addition, our finding that the S688Y mutation in GluN1 significantly increased the τ_w_ of desensitisation in GluN3A-containing receptors is consistent with previous reports showing that structural changes in the GluN1 LBD alter the desensitisation properties of GluN1/GluN3A receptors^20–23,27^. Importantly, our results obtained with hippocampal neurons ‒ which express endogenous GluN2 and GluN3 subunits ‒ support our findings in HEK293 cells; interestingly, however, we found that the change in glycine affinity induced by the S688Y mutation was less profound in neurons compared to HEK293 cells. This discrepancy have several possible explanations, including: (i) the presence of other NMDAR subtypes in hippocampal neurons compared to the subtypes we expressed in HEK293 cells, including triheteromeric^9,62^ and/or GluN3A-containing NMDARs^10,63^; (ii) possible differences in posttranslational modifications such as glycosylation^64^; (iii) possible differences in other proteins that interact with NMDARs^65^, and (iv*)* the use of different concentrations of glutamate (HEK293 cells) and NMDA (hippocampal neurons).

Our finding that the S688Y mutation in GluN1 subunit profoundly reduces the surface delivery of GluN3A-containing NMDARs is consistent with our recent report that the LBD’s sensitivity for glycine is the key factor that regulates the surface delivery of these types of NMDARs^27^. On the other hand, our finding that the S688Y mutation does not appear to affect the surface delivery of GluN1/GluN2A or GluN1/GluN2B receptors differs from a previous report that the D732A mutation in GluN1 affects the trafficking of GluN1/GluN2A receptors^28^; however, this difference may be explained by differences between the D732A and S688Y mutations with respect to changing the conformation of functional NMDAR heterotetramers, thereby affecting the surface delivery of the resulting GluN1/GluN2A receptors. It is interesting to note that the EC_50_ for l-glutamate is also correlated with reduced surface delivery of GluN1/GluN2B receptors^29,66^. Similarly, the NMDARs carrying several pathogenic mutations in the LBDs of GluN2A and GluN2B subunits exhibit clear correlation between the EC_50_ for  l-glutamate and surface expression^40^. For example, the pathogenic E413G mutation in GluN2B subunit profoundly reduced the surface delivery of NMDARs^40^, likely by promoting the unbinding of  l-glutamate and opening of the LBD^67^. On the other hand, other pathogenic mutations within the LBDs of GluN2A and GluN2B subunits revealed no clear correlation between the receptor’s EC_50_ for  l-glutamate and surface expression, suggesting that additional mechanisms than the potency of  l-glutamate regulate the surface delivery of the GluN1/GluN2 receptors^40^.

Our finding that the S688Y mutation in GluN1 reduces NMDA-induced excitotoxicity supports the notion that a pathogenic missense mutation in a GluN subunit can profoundly affect neuronal survival. Nevertheless, an open question is whether this in vitro effect is relevant in vivo, particularly given that specific de novo pathogenic mutations in patients are heterozygous. Moreover, whether the reduction in surface delivery of GluN3A-containing NMDARs is functionally relevant is currently unknown; however, it is interesting to speculate that a reduction in the surface expression of GluN3A-containing NMDARs may alter dendritic spine maturation^68,69^ and may change the neuron’s vulnerability to ischaemic events, given that the GluN3A subunit has been found to reduce neuronal apoptosis^70,71^.

In summary, our results indicate that the functional effects of putative pathogenic mutations in GluN subunits should be examined carefully and systematically using a variety of techniques, particularly given that endogenous NMDARs are comprised of a wide variety of GluN subunit combinations. Nevertheless, our findings provide new insights into the role that the ligand-binding domain in the GluN1 subunit plays in NMDAR trafficking and functioning.

## Methods

### In silico modelling

The structure of the GluN1/GluN2A LBD was obtained from the RCSB Protein Data Bank – PDB ID: 5KCJ^49^ (structure of the human GluN1/GluN2A LBD in complex with GNE6901, resolution 2.09 Å, no outliers according to Ramachandran et al.^72^). The numbering of the amino acid residues provided in the original GluN1/GluN2A receptor downloaded from the Protein Data Bank was revised based on the human full-length GluN1 subunit sequence (PubMed gene ID: 2902, NCBI Reference Sequence (RefSeq): NM_001185091.2) as follows: Q14 = Q405, P125 = P516, T127 = T518, R132 = R523, S181 = S688, V182 = V689, W224 = W731, and D225 = D732. The GluN1/GluN2A LBD structure was prepared using the DockPrep function of UCSF Chimera (v. 1.4). The position of the Y688 residue in the GluN1-S688Y/GluN2A LBD was obtained from the Dunbrack rotamer library as the most likely rotamer. Two structural water molecules were removed from the mutated receptor due to steric reasons. The energy of the ligand/GluN1/GluN2A LBD was minimised using UCSF Chimera (v. 1.4) with 1000 iterations. The wild-type and mutant GluN1/GluN2A LBDs were converted to pdbqt files using AutodockTools (v. 1.5.6)^73^, and the S688 and Y688 residues were set as flexible for docking, while the other amino acid residues were rigid. Three-dimensional structures of ligands/co-agonists were built using Open Babel (v. 2.3.1), minimised using Avogadro (v 1.1.0), and converted to pdbqt file format using AutodockTools^73^. The docking calculations were made using Autodock Vina (v. 1.1.2) with an exhaustiveness value of 8^74^. The visualisation of the receptor-ligand interactions was prepared using the PyMOL Molecular Graphics System, v. 2.0 (Schrödinger LLC, Mannheim, Germany).

### Mammalian expression vectors and lentiviruses

For this study, we used cDNA vectors expressing untagged human GluN1-4a (hGluN1-4a; NCBI RefSeq NM_001270610.1 was modified to the human version by changing the four amino acid residues (N159S, R212K, I267L, M415L) that differ between the rat and human GluN1-4a subunits)^27^, GluN2A (hGluN2A; NCBI Reference Sequence: NM_000833.5), GluN2B (hGluN2B; NCBI RefSeq: NM_000834.5), and GluN3A (hGluN3A; NCBI RefSeq: NM_133445.3) subunits^27,75,76^, GFP-tagged versions of rat GluN2A (GFP-rGluN2A; NCBI RefSeq: NM_012573.3) and GluN2B (GFP-rGluN2B; NCBI RefSeq: NM_012574.1) subunits, and GFP-tagged human GluN3A (GFP-hGluN3A; NCBI RefSeq: NM_133445.3)^27,77^. The YFP-tagged hGluN1-1a subunit (NCBI RefSeq NM_017010.2 was modified to the human version as described above)^76^ was cloned into the FHUGW lentivirus vector containing 20 sense nucleotides in the GluN1 target sequence (gac cgg aag ttt gcc aac ta; with a short hairpin (AAGCTT) and 20 antisense nucleotides cloned downstream of the H1 promoter) to knock down the endogenous GluN1 subunit, as described previously^53^. Silent mutations (gac cgC aaA ttC gcG aac ta; the mutated nucleotides are indicated in capital letters) were introduced in order to generate shRNA-resistant versions of YFP-hGluN1-1a and YFP-hGluN1-1aS688Y . All mutations were introduced using the Quick-Change site-directed mutagenesis kit (Agilent Technologies), and the full GluN-coding sequences were confirmed by sequencing. Lentiviruses expressing YFP-hGluN1-1a and YFP-hGluN1-1a-S688Y were prepared in HEK293T cells by co-transfecting the FHUGW lentiviral vector with Δ8.9 and VSVG as described previously^53^, and supernatants containing the viral particles were harvested 60 h after transfection.

### Mammalian cell culture and primary hippocampal neurons

Human embryonic kidney 293 (HEK293) cells were maintained in Opti-MEM I media containing 5% (v/v) foetal bovine serum (FBS; Thermo Fisher Scientific). The cells were transfected using Lipofectamine 2000 (Thermo Fisher Scientific) as described previously^64^. After transfection, the HEK293 cells used for electrophysiology were dissociated with trypsin; the cells used for microscopy and biochemistry were cultured without the trypsinisation step in culture media containing 1% FBS, 20 mM MgCl_2_, 1 mM D,L-2-amino-5-phosphonopentanoic acid, and 3 mM kynurenic acid (to prevent cell death caused by excessive activation of NMDARs). The cells were plated on poly-L-lysine-coated glass coverslips and were used 24–48 h after transfection.

All procedures involving the use of laboratory animals were performed in accordance with the European Communities Council Directive November 24, 1986 (86/609/EEC) and animal care guidelines approved by the Institute of Experimental Medicine CAS Animal Care Committee. Primary cultures of hippocampal neurons were prepared from embryonic day 18 Wistar rats^53^. In brief, the hippocampi were removed, placed in cold Hank's Balanced Salt Solution containing 10 mM HEPES (pH 7.4), and incubated for 20 min at 37 °C in dissection media containing 0.1 mg/ml DNase I and 0.05% trypsin (Merck). The cells were then dissociated by trituration through a fire-polished glass pipette and resuspended in plating medium consisting of Neurobasal media with B-27 supplement and  l-glutamine (Thermo Fisher Scientific). The cells were grown at a density of approximately 2 × 10^4^ cells per cm^2^ on dishes coated with poly- l-lysine (Sigma). The neurons were fed every 7 days with fresh plating media, infected with lentiviruses after 5–7 in culture, and used for experiments 10 days after infection.

### Electrophysiology

Whole-cell patch-clamp recordings were performed using an Axopatch 200B amplifier (Molecular Devices) at room temperature using intracellular recording solution containing (in mM): 125 gluconic acid, 15 CsCl, 5 BAPTA, 10 HEPES, 3 MgCl_2_, 0.5 CaCl_2_, and 2 ATP-Mg salt (pH adjusted to 7.2 with CsOH)^75^. Glass patch pipettes (3–6 MΩ tip resistance) were prepared using a model P-1000 micropipette puller (Sutter Instrument Co.). A microprocessor-controlled multi-barrel rapid perfusion system (with a time constant for solution exchange around the cell of approximately 20 ms) was used to apply the extracellular recording solution (ECS)^75,78^. The control ECS contained (in mM): 160 NaCl, 2.5 KCl, 10 HEPES, 10 glucose, 0.2 EDTA, and 0.7 CaCl_2_ (pH adjusted to 7.3 with NaOH)^79^. When recording hippocampal neurons, the ECS also contained 1 µM tetrodotoxin (TTX) and 10 µM bicuculine. pCLAMP 9 software (Molecular Devices) was used to record and analyse the NMDAR-induced currents recorded in voltage-clamp mode at a membrane potential of − 60 mV. The normalised steady-state and peak concentration–response data for each recording were best-fitted using the following equation: *I* = *I*_*max*_/(1 + (EC_50_/[Agonist])^*h*^), where *I*_*max*_ is the maximum peak current in response to agonist, EC_50_ is the agonist concentration (in μM) that elicited the half-maximal response, [Agonist] is agonist concentration (in μM), and *h* is the apparent Hill coefficient. Data were fitted using SigmaPlot 14.0 (Systat Software, Inc.).

### Surface expression analysis

HEK293 cells grown in 12-well plates were transfected with GluN subunit combinations using Lipofectamine 2000 as described previously^27^. Live-cell labelling of GFP/YFP-GluN subunits at the cell surface was performed using phosphate-buffered saline (PBS) containing 0.2% bovine albumin serum (BSA) and the rabbit anti-GFP primary antibody (1:1000; Merck) followed by an anti-rabbit antibody conjugated to either Alexa Fluor 555 (1:1000; Thermo Fisher Scientific; for HEK293 cells) or Alexa Fluor 647 (1:1000; Thermo Fisher Scientific; for hippocampal neurons)^27,53,77^. The stained cells were then fixed in 4% paraformaldehyde (PFA) in PBS for 20 min. To label the total pool of GluN subunits, fixed cells were permeabilised with 0.25% Triton X-100 (Serva) in PBS for 5 min. The cells were then labelled in PBS containing 0.2% BSA (HEK293 cells) or 3% normal goat serum (hippocampal neurons) containing the mouse anti-GFP antibody followed by an anti-mouse antibody conjugated to Alexa Fluor 488 (1:1000; Thermo Fisher Scientific)^27,53,77^. The labelled cells were mounted using ProLong Gold Antifade reagent (Thermo Fisher Scientific) and imaged using an Olympus FV10i confocal microscope with a 60x/1.35 oil immersion objective (for HEK293 cells) or a Leica SP8 confocal scanning microscope with a 63x/1.40 oil immersion apochromatic objective (for hippocampal neurons). The *z*-stack for all images was 0.3 µm, and the resolution was 512 × 512 pixels and 2048 × 2048 pixels for HEK293 cells and hippocampal neurons, respectively. The surface and total fluorescence intensities of HEK293 cells were analysed on whole-cell areas using ImageJ 1.52 N (National Institutes of Health, Bethesda, MD). For hippocampal neurons, the fluorescence intensity of the total and surface signals was analysed for 5 separate 10-µm segments of secondary or tertiary dendrites per neuron^27,53,80^. Prior to the intensity analysis, a *z*-stack projection was made with maximal intensity from the bottom of the cell to the top of the cell.

### Biochemistry

For the surface biotinylation assay^81^, cultured hippocampal neurons were labelled with 0.5 mg/ml EZ-Link Sulfo-NHS-LC-biotin (Thermo Fisher Scientific) in PBS++ solution (0.01 M PBS, pH 7.4, supplemented with 1 mM MgCl_2_ and 0.1 mM CaCl_2_) for 20 min at 4 °C with gentle mixing. The remaining biotin was quenched with PBS++ containing 50 mM glycine. The biotinylated neurons were lysed, centrifuged, and the supernatant was incubated with 20 µl of streptavidin-agarose beads (Thermo Fisher Scientific) for 3 h at 4 °C. After washing the beads 3 times with lysis buffer, the bound proteins were eluted and analysed using western blot.

For PSD fractionation in neurons^82^, cultured cortical neurons were homogenised in hypotonic buffer (10 mM Tris–HCl, pH 8.0, 10 mM KCl, 0.1 mM EDTA) and passed through a 23-G needle 20 times to disrupt the plasma membrane. Sucrose was then added to a final concentration of 0.32 M. The nuclear pellet and any remaining intact neurons were removed by centrifugation at 800×*g* for 5 min. The supernatant was then centrifuged at 18,000×*g* for 20 min to obtain a crude synaptosome pellet. This pellet was resuspended in TNE buffer (50 mM Tris–HCl, pH 8.0, 150 mM NaCl, 2 mM EDTA) containing a protease inhibitor cocktail (Roche) and solubilised with 1% Triton X-100 for 5 min on ice. The samples were then centrifuged at 18,000×*g* for 15 min, and the resulting pellet was further solubilised in TNE buffer containing 1% SDS. After the insoluble material was removed, the supernatant was harvested as the PSD fraction.

### Calcium imaging

Hippocampal neurons were pre-incubated for 30 min with Fura-2, AM (2.5 μM, Thermo Fisher Scientific) and the non-ionic detergent Pluronic F-127 (0.05% (w/v) in DMSO, Merck), and imaged in ECS containing 1 µM TTX and 10 µM bicuculine. The Fura-2 signal was captured using an inverted AxioObserver D1 microscope controlled with ZEN 2012 software (Zeiss) and equipped with a CCD camera and Lambda-DG4 fast illumination system (Sutter Instruments, Novato) for excitation at 340 and 380 nm. The fluorescence intensity of the Fura-2 emission was measured at 510 nm as a ratio of signals obtained after excitation at 340 and 380 nm. Data was sampled every 500 ms during the Ca^2+^ imaging. The cells were continuously perfused with ECS at 37 °C, and solutions were exchanged using a multiple capillary perfusion system consisting of a computer-controlled multichannel peristaltic pump (Reglo ICC, Ismatec). Traces of individual cells expressed as the F340/F380 ratio were horizontally aligned by their baseline and analysed using Matlab 2019b (MathWorks). An average trace was calculated as the mean of all recorded cells, with the shaded area corresponding to the standard error of the mean (SEM).

### NMDA-induced excitotoxicity

Excitotoxicity was induced as described previously^55^. In brief, the cultured neurons were incubated overnight in 10% MEM (Thermo Fisher Scientific) and 90% salt-glucose (SG) medium containing 114 mM NaCl, 0.219% NaHCO_3_, 5.292 mM KCl, 1 mM MgCl_2_, 2 mM CaCl_2_, 10 mM HEPES, 30 mM glucose, 0.5 sodium pyruvate, and 0.1% phenol red. The following day, the media was replaced with 100% SG and the indicated concentrations of agonists/co-agonists were added to the neurons. After 1 h, the medium was replaced with 10% MEM and 90% SG medium; 23 h later, the neurons were stained with Hoechst 33,342 (5 µM, Molecular Probes) for 30 min, fixed in 4% PFA, and the YFP-hGluN1 subunits were labelled with rabbit anti-GFP primary and anti-rabbit Alexa Fluor 488 secondary antibodies as described above. The images (1024 × 1024 pixels with a pixel size of 1.243 × 1.243 µm, covering a field of 1272 × 1272 µm) were acquired using an Olympus FV10i confocal microscope with a 60x/1.35 oil immersion objective; the following three images were obtained for each field of view: the YFP signal (for infected cells), Hoechst 33,342 (to stain the nuclei), and a widefield image. Nuclear area was measured using ImageJ software (v. 1.52p), and custom-made macro scripts were used to automatically measure only the nuclei of infected cells (identified by YFP expression). We plotted all of the measured nuclei from a single experiment as a histogram containing control (with mostly non-pyknotic cells) and each tested conditions (mostly with a mixture of pyknotic and non-pyknotic cells). The histogram contained two clearly distinguishable groups corresponding to pyknotic cells and non-pyknotic cells. The MatLab function “fitgmdist()” was used to fit the histograms with two Gaussian functions. The data for each condition were then passed with the estimated Gaussian mixture model parameters in the MatLab function “cluster()”, which estimated the posterior probability of each nuclear area belonging to one of the distributions and classified the cells into two groups. The ratio of the number of cells in each group was then used to estimate the effects of mixing the two distributions and is expressed as the ratio of pyknotic and non-pyknotic cells. The percentage of pyknotic cells in each condition was then calculated as the number of cells classified as pyknotic divided by the total number of cells in that condition.

### Statistical analysis

Except where indicated otherwise, all summary data are presented as the mean ± SEM. Group differences were analysed using the Student’s *t*-test or a one-way ANOVA followed by Dunnett’s post hoc text or Shapiro–Wilk normality test. Data were analysed using SigmaStat 3.5 (Systat Software, Inc.), and differences with a *p* value < 0.05 were considered significant.

## Supplementary information

Supplementary Information.

## Data Availability

All materials will be provided promptly upon request without undue qualifications for material transfer agreement.
