# Supplementary material for: The pathogenic S688Y mutation in the ligand-binding domain of the GluN1 subunit regulates the properties of NMDA receptors
Source: Sci Rep. 2020 Oct 29;10:18576. doi: 10.1038/s41598-020-75646-w (PMC7596085; doi:10.1038/s41598-020-75646-w)
Supplement: Supplementary file 1 — Supplementary Information. [file 41598_2020_75646_MOESM1_ESM.pdf]

Supplementary data

The pathogenic S688Y mutation in the ligand-binding domain of the GluN1 subunit regulates the properties of NMDA receptors

Kristyna Skrenkova[1,#], Jae-man Song[2,#], Stepan Kortus[1], Marharyta Kolcheva[1,3], Jakub Netolicky[1,3], Katarina Hemelikova[1], Martina Kaniakova[1], Barbora Hrcka Krausova[1], Tomas Kucera[4], Jan Korabecny[4,5], Young Ho Suh[2*], Martin Horak[1*]

[1] Institute of Experimental Medicine of the Czech Academy of Sciences, Praha, Czech Republic

[2] Department of Biomedical Sciences, Neuroscience Research Institute, Seoul National University College of Medicine, Seoul, South Korea

[3] Department of Physiology, Faculty of Science, Charles University in Prague, Prague, Czech Republic

[4] Department of Military Medical Service Organisation and Management, and Department of Toxicology and Military Pharmacy; Faculty of Military Health Sciences, University of Defence, Hradec Kralove, Czech Republic

[5] Biomedical Research Centre, University Hospital Hradec Kralove, Hradec Kralove, Czech Republic

# contributed equally

* Address correspondence and reprint requests to:

Martin Horak, Department of Neurochemistry, Institute of Experimental Medicine of the Czech Academy of Sciences, Videnska 1083, 14220 Prague 4, Czech Republic; E-mail: martin.horak@iem.cas.cz

Young Ho Suh, Department of Biomedical Sciences, Seoul National University College of Medicine, 103 Daehak-ro, Jongno-gu, Seoul 03080, South Korea; E-mail: suhyho@snu.ac.kr

Supplementary Figure S1

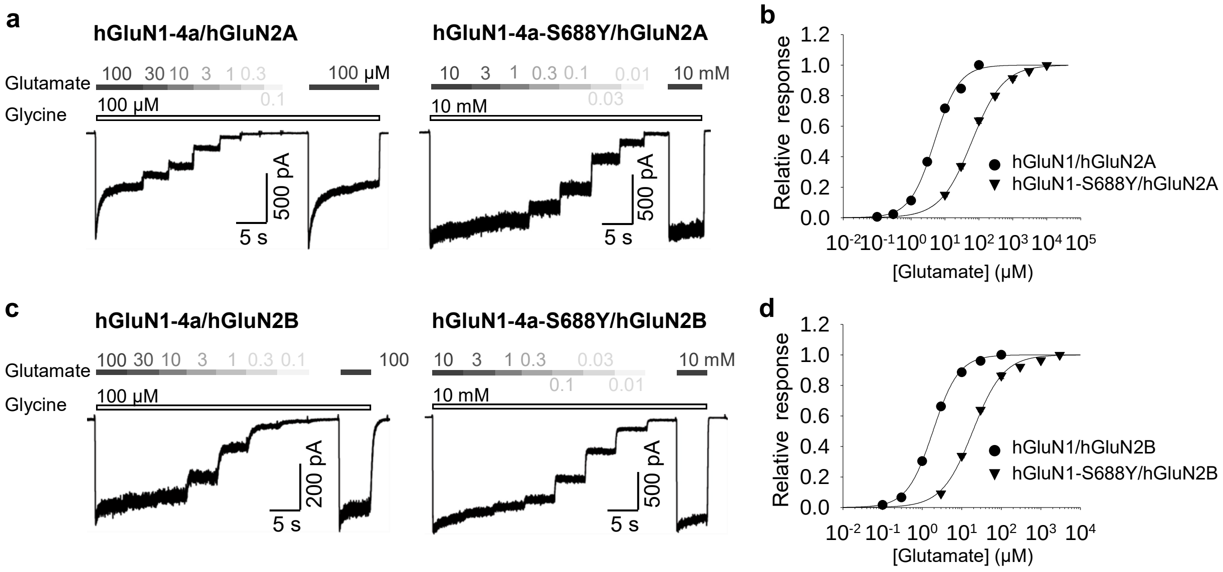

Figure S1. The S688Y mutation in GluN1 alters the functional properties of NMDARs. (a,c) Representative whole-cell patch-clamp recordings of HEK293 cells expressing the indicated hGluN1-4a and hGluN2 subunits. Currents were elicited by applying glutamate at the indicated concentrations (in µM) in the presence of 100 µM or 10 mM glycine; where indicated, 10 µM 7-chlorokynurenic acid (KYNA) was applied to block. (b,d) Steady-state concentration-response curves measured in cells expressing the indicated hGluN1-4a and hGluN2 subunits. Each data point represents the normalised steady-state current (±SEM). The EC50 values, Hill coefficients (h) and the numbers of recorded cells (n) were following: hGluN1-4a/hGluN2A (EC50 = 4.94 ± 0.39 µM; h = 1.20 ± 0.04; n = 7), hGluN1-4a-S688Y/hGluN2A (EC50 = 62.29 ± 4.86 µM; h = 0.93 ± 0.05; n = 8), hGluN1-4a/hGluN2B (EC50 = 1.89 ± 0.05 µM; h = 1.33 ± 0.03; n = 6), hGluN1-4a-S688Y/hGluN2B (EC50 = 19.18 ± 1.29 µM; h = 1.11 ± 0.05; n = 8).

Supplementary Figure S2

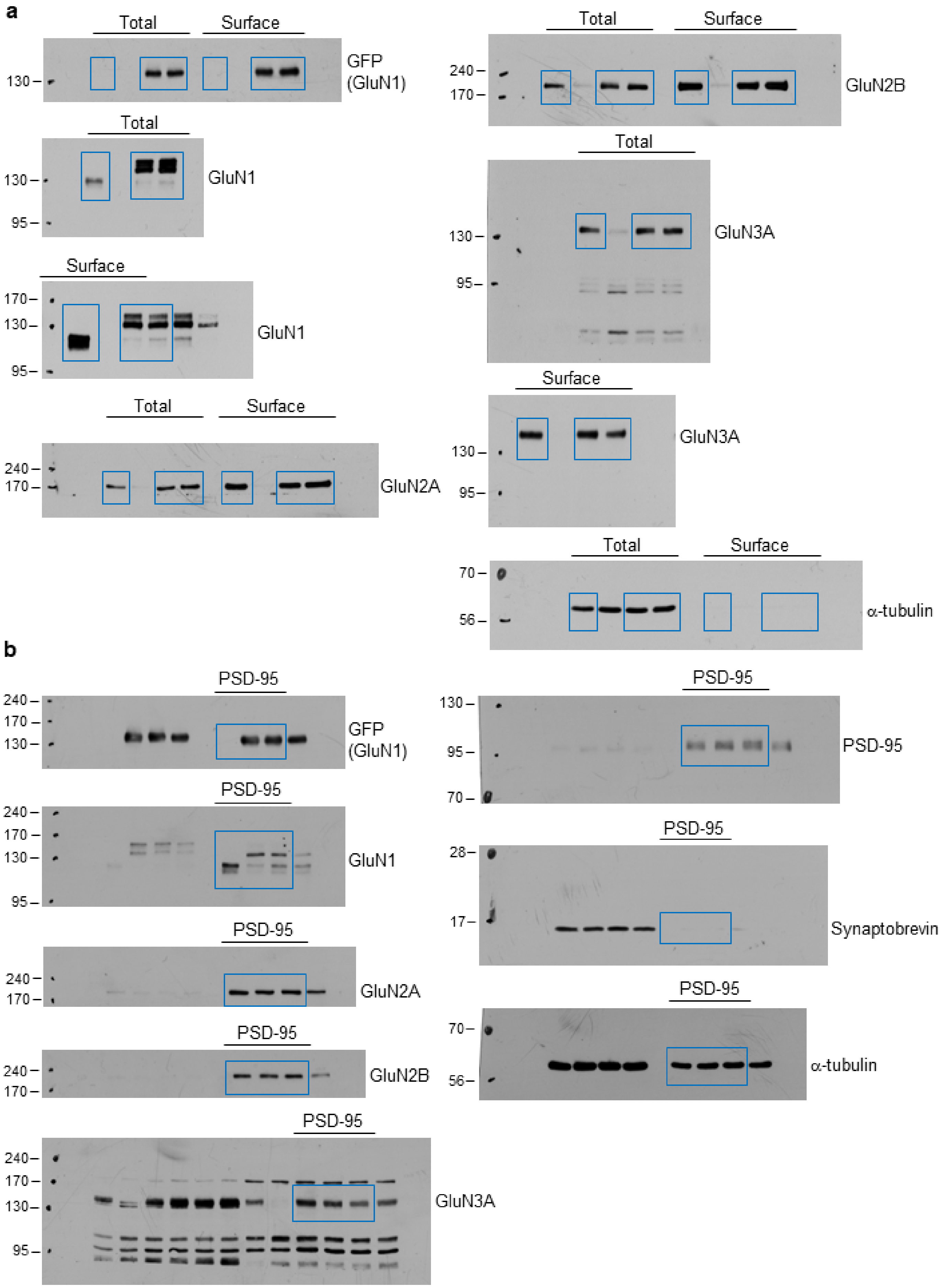

Figure S2. The blots for Figure 4a (a) and Figure 4c (b).
